# Emission characteristics of ultrafine particles from bare and Al_2_O_3_ coated graphite for high temperature applications

**DOI:** 10.1038/s41598-020-71424-w

**Published:** 2020-09-03

**Authors:** S. K. Yadav, P. Shukla, Manish Joshi, Arshad Khan, A. Kaushik, Ajit Kumar Jha, B. K. Sapra, R. S. Singh

**Affiliations:** 1 Department of Mechanical Engineering, Indian Institute of Technology (BHU), Varanasi, 221005 India; 2 Department of Chemical Engineering, Indian Institute of Technology (BHU), Varanasi, India; 3 Radiological Physics and Advisory Division, Bhabha Atomic Research Center, Mumbai, India; 4 High Temperature Reactor Section, Bhabha Atomic Research Center, Mumbai, India

**Keywords:** Environmental impact, Nuclear energy

## Abstract

Owing to its exceptional properties at high temperature, graphite is used in several applications such as structural material and fuel block in high temperature nuclear reactors. Air ingress is one of the serious safety concerns in these reactors. Oxidation of graphite leading to increased porosity affects its mechanical strength and may lead to core collapse resulting in a severe accident. During such a scenario, generation of graphite particles could be the main hazard. Once generated, these particles often in fine and ultrafine sizes, may carry radioactivity to large distances and/or for long times. These particles owing to their higher surface to volume ratio possess an additional inhalation hazard. Ultrafine particles have the potential to enter into respiratory tract and cause damage to body organs. Coating of graphite components is preferred to reduce the oxidation induced damages at high temperatures. In the present work, effect of alumina (Al_2_O_3_) coating on the emission characteristics of particles from graphite under high temperature conditions has been investigated. Bare and Al_2_O_3_ coated graphite specimens were heated within a closed chamber at varying temperatures during these experiments. Temporal evolution of concentrations of gases (CO and CO_2_) and particles were measured. The results reveal that Al_2_O_3_ coating on the graphite delayed the oxidation behavior and the structure of graphite remained largely intact at high temperatures. A significant reduction in aerosol formation and CO emission was also noticed for the coated specimens.

## Introduction

Graphite is used as a structural material in high temperature reactors (HTRs) owing to its excellent properties at high temperatures^1,2^. Properties such as low neutron absorption, high strength, low thermal expansion coefficient, high thermal conductivity etc. make it a preferred choice for nuclear applications^3–5^. Graphite has been tested at all operating conditions of HTRs and has been found to fulfill the operational demands at high temperatures. In HTRs, graphite is used as a moderator, reflector and fuel block/tube. It is also used as electrodes^6^ and refractories in high temperature material processing applications^7^. Ingress of foreign media, i.e., air and water into the primary circuit during normal and off-normal conditions has been highlighted as a safety issue for HTRs^8^. Such ingress affects surface characteristics and properties of graphite components, particularly at high temperatures. One of the concern in these conditions is the ‘oxidation of graphite’ and the consequent change in structural characteristics^9–11^. Loss of mechanical strength of fuel block and core collapse leading to severe accident is a part of ‘Probabilistic Safety Assessment’ for HTRs. In such postulated accident scenario in prismatic block type HTRs, graphite oxidation at high temperature may result in the release of particles to the containment and to the atmosphere. Expected to be formed by nucleation, these particles grow to ultrafine (< 100 nm geometric diameter) sizes and may act as a carrier for fission products. Due to significantly longer residence time and larger interfacial activeness, they possess inhalation hazard after spreading the radioactivity to a larger domain^12–14^. In the past, transport of particles over thousands of kilometers was associated with Chernobyl nuclear accident^15,16^. For pebble bed HTRs, the source of carbonaceous dust is the abrasion of fuel pebbles. These particles are shown to be generated in coarse (1–6 µm) size-ranges^17^. Once generated, formation and evolution characteristics of particles become the key to determine their life and fate^18^. The transport and deposition of graphite particles and fission products in different reactor components has been studied in past^19–21^.

Due to wide spread usage of graphite in present and future designs of HTRs, researchers focus on different issues which could be instrumental in improving the desired characteristics at high temperature. In the past, authors have investigated the oxidation behaviour and the effect of controlling factors on the same^22–24^. Oxidation reaction was observed to be controlled by chemical kinetics at lower temperature and in-pore diffusion at higher temperature^25–27^. Arrhenius plots have been used for the estimation of activation energies during the oxidation process^28,29^. The transition temperature for in-pore diffusion depends on impurities, density and microstructure of graphite^30^. Above transition temperature (typically within the range of 600–900 °C), the oxidation rate of graphite increases resulting in significant weight loss^26,31^.The oxidation rate of graphite also gets limited by the number of available reactive surface area sites within the graphite microstructure^32^.

Coating of different type of ceramics e.g. Al_2_O_3_, SiC, MoSi_2_, ZrB_2_, SiO_2_, Si3N_4_, B_4_C, etc. on the graphite surface has been explored in order to cut diffusion channel for oxygen to attack the substrate^33–40^. Out of these coating options, MoSi_2_ and B_4_C coatings are expected to severely affect the neutronic behavior of the reactor. However, due to limited volume fraction, other coating options are not suspected to modify the neutronic behavior of the core significantly. Aluminum has been widely used in nuclear industry as fuel clad and other structural material^41^. Al_2_O_3_ coating seems to be one of the most promising option for the protection of graphite components due to its strength retention, high melting temperature and superior oxidation resistance. Al_2_O_3_ coated graphite has lower porosity, higher strength and stability than uncoated graphite^42–44^. However, maintaining the purity of coating is crucial as the impurities may adversely affect the neutronic behavior of the reactor. Also, the effect of neutron irradiation on thermal diffusivity could be detrimental for large volume fraction of coating^45^.

Although the role of coating on the oxidation behavior of graphite has been investigated, little information is available about particle generation. The possibility of the generation of ultrafine particles even with a negligible oxidation induced structural change at high temperature cannot be ruled out. Such an emission cannot be characterized in terms of weight loss or the shape modifications, but can be characterized on the basis of real time number concentration measurements. This study focuses on studying the effect of Al_2_O_3_ coating on the generation of ultrafine particles at different temperature conditions. Experiments have been conducted in a specially fabricated high temperature aerosol sampling facility. Results in terms of particle emission characteristics (number concentration and size distribution) have been corroborated with gaseous (CO, CO_2_) emission measurements and surface characterization (of graphite specimen) for gaining valuable insights related to coated graphite behaviour at high temperature.

## Material characterization and methodology

### Experimental setup

**Figure 1:**
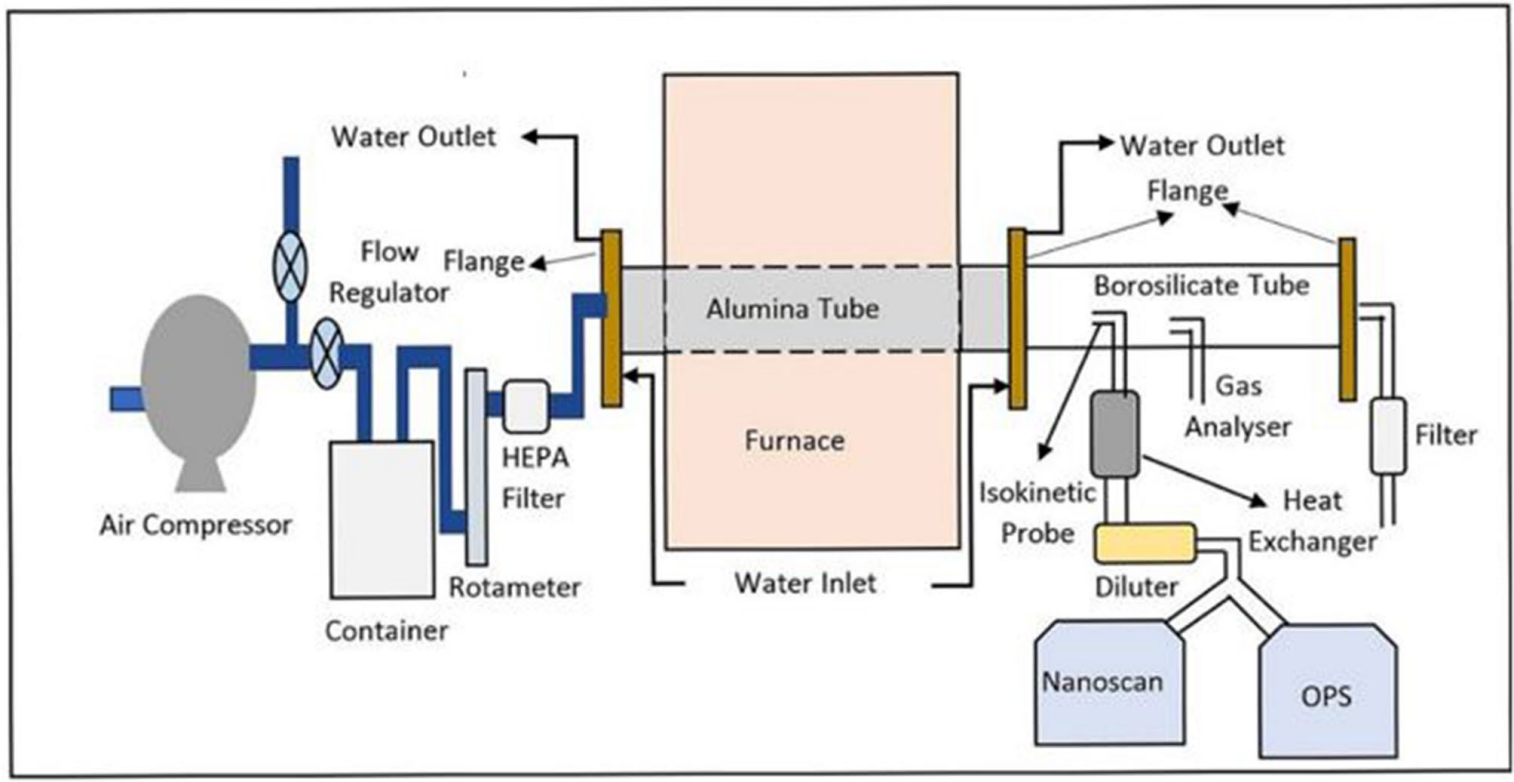
Schematic diagram of the experimental setup^46^.

An experimental facility has been developed to study and interpret the phenomenological aerosol emission characteristics while subjecting graphite to high temperature conditions. Schematic diagram of the facility and associated experimental set-up is shown in Fig. 1. The core component of this facility is a ‘tubular furnace’. High purity air from an air compressor is supplied to the furnace after passing it through a high efficiency particulate air filter (99.97% collection efficiency for 0.3 µm). The flow rate can be controlled and measured by a rotameter attached to the compressor assembly. The ‘test material’ can be placed in the furnace tube which can then be subjected to target temperature. The clean air acts as an oxidant as well as a carrier gas, transporting the emissions from the test material to the other end of the tube. The oxidation of graphite in air ingress scenario depends primarily on two factors viz. the amount of oxygen and the core heat balance. For the worst case scenario (loss of coolant), ingress of air dominates the concentration of other coolant gases. This is also relevant/applicable to localized regions of the structure (close to the leaks) where air comes in direct contact of graphite components. The downstream side of the facility consists of an iso-kinetic probe, borosilicate tube, double pipe heat exchanger and the aerosol instrumentation. Presence of iso-kinetic probe, aerosol diluter and heat exchanger limit the temperature and concentration of aerosol stream below the working range of the aerosol instrumentation. The exit air from the diluter passes through a flow splitter and ultimately to aerosol measurement instruments comprising of a Scanning Mobility Particle Sizer (Nanoscan SMPS 3910, Make- TSI, USA) and Optical Particle Sizer (OPS 3330, Make- TSI, USA). Total number concentration and number size distribution in the size range of 10 nm–10 µm can be measured by the combination of these sizers. Aerosol-laden air is also analyzed by a gas analyzer (Digas 444, Make-AVL, India) by inserting a probe in the Borosilicate tube. Details of this facility have also been discussed elsewhere^46^.

### Material and its characterization

**Figure 2:**
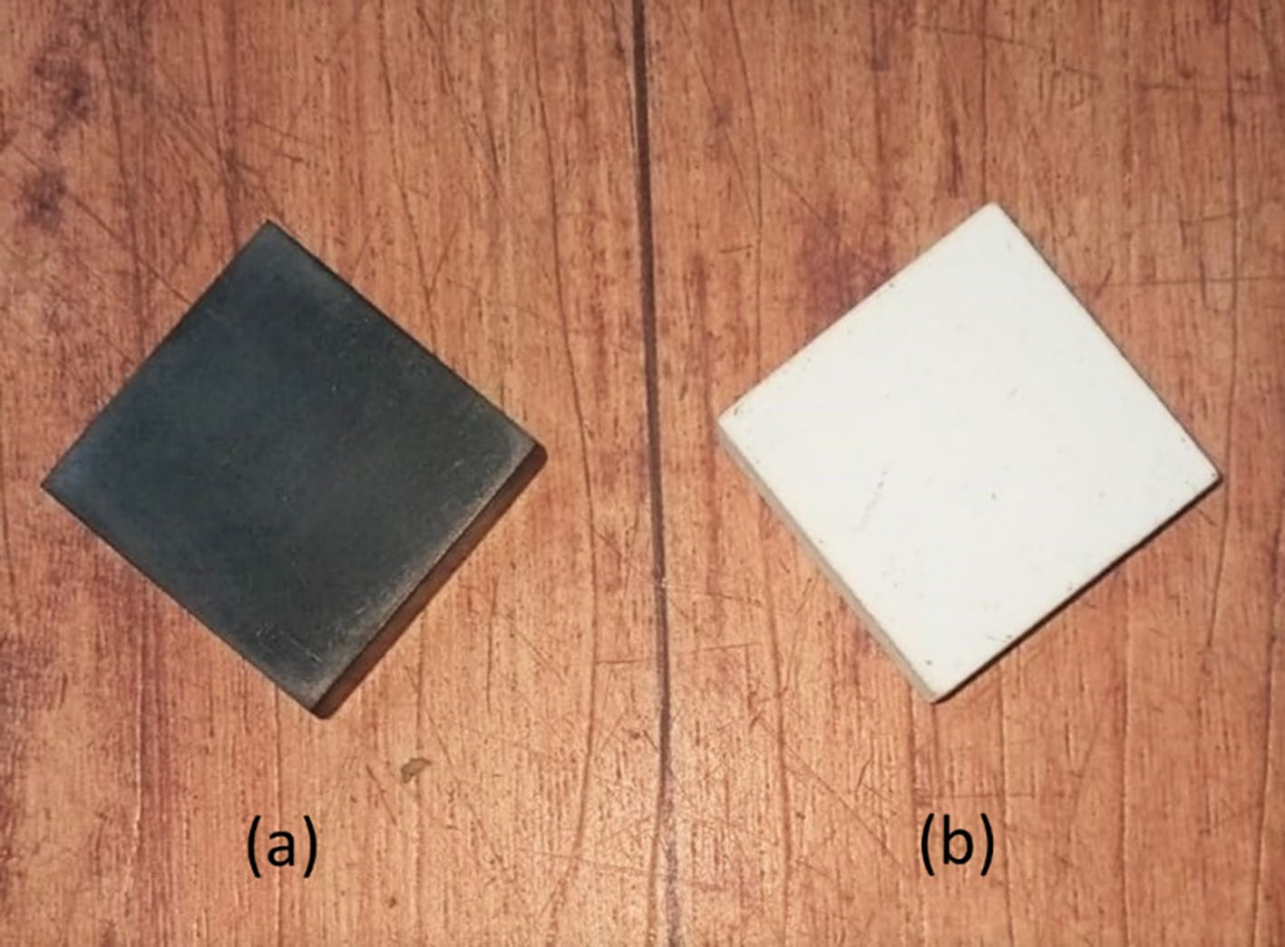
Picture of (**a**) ‘bare graphite’ and (**b**) ‘coated graphite’.

The high-density isotropic ultrafine grain graphite specimens with 99.96% carbon content by weight were cut into specimens of size 25 mm × 25 mm × 5 mm, each having a weight of approximately 7.0 g. Properties of the above mentioned grade of graphite are shown in Table 1. Same grade of graphite was also used for the preparation of reference graphite material in past^47^. As can be seen from Table 1, equivalent boron content of the specimens used in this work was not lesser than 10 ppm—an essential characteristic for qualifying it as ‘nuclear grade graphite’^48^. However, this slight elevation of boron content is not expected to modify the aerosol generation characteristics significantly as other properties of these specimens conform to the requirement of a ‘nuclear grade graphite’^48^**.** These specimens were then cut in specialized in-house graphite workshop. Before actual experiments, the specimens were cleaned with liquid nitrogen and subsequently dried. This was essential to remove any inadvertent contaminant/deposit on the surface and within micro-pores/micro-cracks. These cleaned pieces are referred to as ‘bare graphite’ in the following sections. For obtaining ‘coated specimens’, graphite lapping was performed by Al_2_O_3_ powder with grain size of 12–16 μm. The powder deposited on bare graphite by plasma spraying consisted of ≈ 97% Al_2_O_3_ with minor fractions of SiO_2_ (≈ 2%) and Na_2_O (≈ 0.75%). The process was controlled so as to maintain the coating thickness as nearly uniform (100 ± 10 µm). These Al_2_O_3_ deposited specimens are referred to as ‘coated graphite’ in the following sections. In general, a higher thickness of coating (increased volume fraction) may affect the neutronic balance and thermal diffusivity while lesser thickness impacts the oxidation shielding. Lesser electrical insulation resistance was also linked to lesser thickness of plasma sprayed Al_2_O_3_ coating^49^. A typical picture of both types of specimens i.e. ‘bare graphite’ and ‘coated graphite’ has been depicted in Fig. 2. Two pieces of each type were used in each experiment performed in this work.

**Table 1:** Properties of graphite specimen.

Properties	Value/details
Mode of fabrication	Isostatically pressed
Equivalent Boron content	≈13 ppm
Density	1.82 g cm^−3^
Porosity	10.53%
Compressive strength	113 MPa
Tensile strength	33 MPa
Flexural strength	40 MPa
Thermal conductivity	93 W/mK

### Experimental conditions

The bare graphite and coated graphite specimens were exposed to high temperature conditions under this work. The experiments were performed at five different temperatures i.e. 500 °C, 600 °C, 700 °C, 800 °C and 900 °C. The upper limit for the test temperatures was due to the stability issues of furnace near the threshold working temperatures. Although the response of coating at core realistic temperatures cannot be investigated, the effect on transition temperature (if any) can be probed. In such a case, the data can be used to supplement engineering safety features in order to inhibit the propagation of accident induced damages. Flow rate of air was maintained at 25 Lmin^−1^ during all sets of experiments. As mentioned earlier, aerosol and gas concentration in the exit air were measured with the combination of aerosol sizers and gas analyzer, respectively. Relatively negligible number concentration of particles was measured by OPS. As the main aim of this work is linked with the number characteristic features, results from only Nanoscan (≈ 10–350 nm) are presented and discussed in this work. ‘Background aerosol number concentration’ was also measured for the set-up. This concentration pertains to the conditions when the furnace is heated without inserting the graphite specimen and corrects the data for any suspected leakage in the set-up or leftover contribution from previous runs. The background concentration was measured as nearly uniform at ≈10^3^ cm^−3^ during the experiments. Each set of experiment was performed at least 3 times to ensure the reproducibility of the obtained results before making any interpretation.

### Experimental procedure

At the start of each experiment, the furnace tube and specimen boat were properly cleaned using a high-flow air jet. The normal air flow was then initiated and the monitoring of aerosol number concentration started. If number concentration was found to be higher than 10^3^ cm^−3^, the set-up was re-cleaned. Otherwise, the furnace was switched on to raise the temperature 100 °C more than the test temperature. This was done to ensure that any combustible material or residues of a previous experimental run get vaporized and purged from the set-up. The furnace was then cooled up to the required test temperature. Subsequently, entrance of the furnace was opened and the specimens placed on the boat were inserted. The furnace was closed after placing the specimen boat at the centre of the tube. The temperature was kept constant for two hours and the particle number and gas concentration at the exit ports were measured. After the experiment, furnace was allowed to cool down to the room temperature. The residual specimens were removed for further analyses.

### Consent for publication

The Authors declare that this manuscript is original, has not been published before and is not currently being considered for publication elsewhere. We confirm that we have given due consideration to the protection of intellectual property associated with this work and that there are no impediments to publication, including the timing of publication, with respect to intellectual property. In so doing we confirm that we have followed the regulations of our institutions concerning intellectual property.

### Ethical approval

We confirm that the manuscript has been read and approved by all named authors and that there are no other persons who satisfied the criteria for authorship but are not listed. We further confirm that the order of authors listed in the manuscript has been approved by all of us.

## Results and discussion

The main aim of Al_2_O_3_ coating on the graphite surface is to avoid/delay the oxidation process at high temperature. This is expected to affect the transition temperature and structural strength of the specimen. In the present work, we focused on measuring and interpreting the role of coating towards the generation of particles from graphite surface at high temperatures. Size, concentration and shape of particles are the main parameters for the measurement and interpretation of formation and growth characteristics. Graphite particles in the context of HTRs have been shown to possess irregular shapes^50–52^. This section presents the results in terms of particle generation characteristics (size and number concentration features), trace gaseous emission and surface characterization of residual specimen after oxidation, respectively.

### Particle generation characteristics

**Figure 3:**
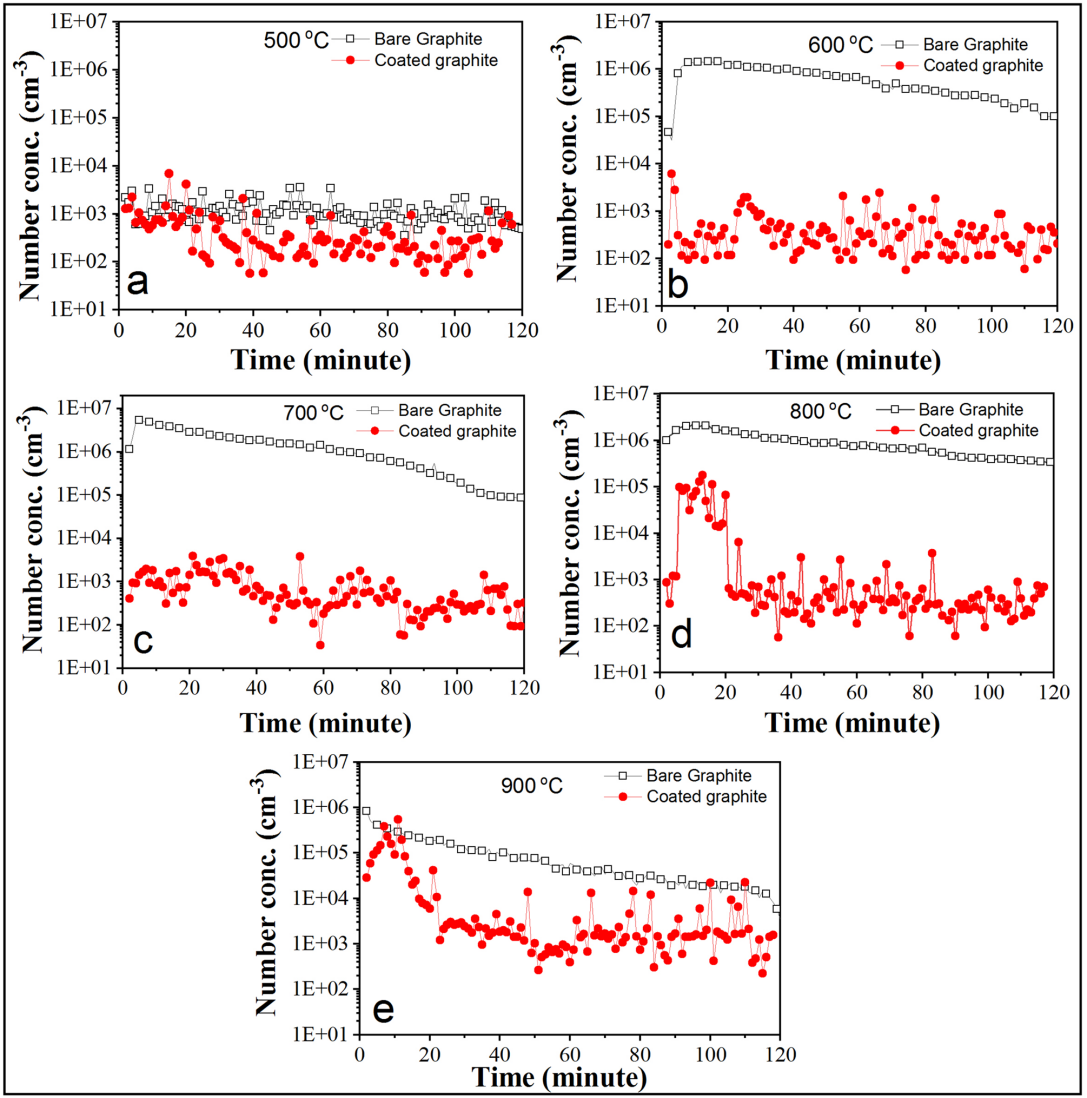
Particle generation in bare and coated graphite at different temperature.

The total number concentration of particles generated due to the oxidation of bare graphite and coated graphite at various constant temperatures is plotted against time and shown in Fig. 3 for one of the experiments. For the temperatures showing oxidation induced signatures, a peaking behavior is observed for the number concentration profile. In the beginning of the oxidation for bare graphite as well as coated graphite specimens, the particle generation was relatively higher. However, with continued heating at constant temperature, the particle emission started declining for all the cases. In the case of heating of bare graphite at 500 °C, negligible particle generation (above background number concentration) was observed. At 600 °C, number concentration was seen to be rising rapidly at initial times, decreasing slowly but steadily afterwards. The peak number concentration measured for this case was 1.4 × 10^6^ cm^−3^. A similar kind of behavior was followed by the number concentration profile at 700 °C. For this case, peak number concentration was found to be highest at 5.3 × 10^6^ cm^−3^ among the tested temperatures. After 700 °C, peak number concentration was found to be lesser relatively. As will be seen later, the behavior of particle emission matched with that of emission of CO gas. This means that the particle formation is linked with the presence of incomplete combustion products. For higher temperatures, CO concentration reduces with a simultaneous increase in CO_2_ gas concentration. Overall, the peak number concentration was found in the range of 0.8 × 10^6^–7.8 × 10^6^ cm^−3^ for the entire set of experiments. The plausible mechanism of particle generation during graphite oxidation have been discussed elsewhere, qualitatively^53^.

When coated graphite was heated for the same experimental conditions, particle generation was not observed above background number concentration till 700 °C. This means that the coating successfully acts as a barrier to the diffusing oxygen preventing the structural integrity of the graphite specimen. However, considerable particle generation was observed during initial stages of heating at 800 °C indicating the onset of pore generation in Al_2_O_3_ coating. With further increase in temperature to 900 °C, sporadic increase of number concentration at initial times continued. This is again an indication of increasing diffusional influx of oxygen at high temperatures, probably due to the increase in porosity and pore size of the exposed specimen. It can also be seen in Fig. 3d–e that the generation of particles from the coated graphite surface occurred only for approximately 20 min of continuous heating after which the number concentration decreased back to the background levels.

**Figure 4:**
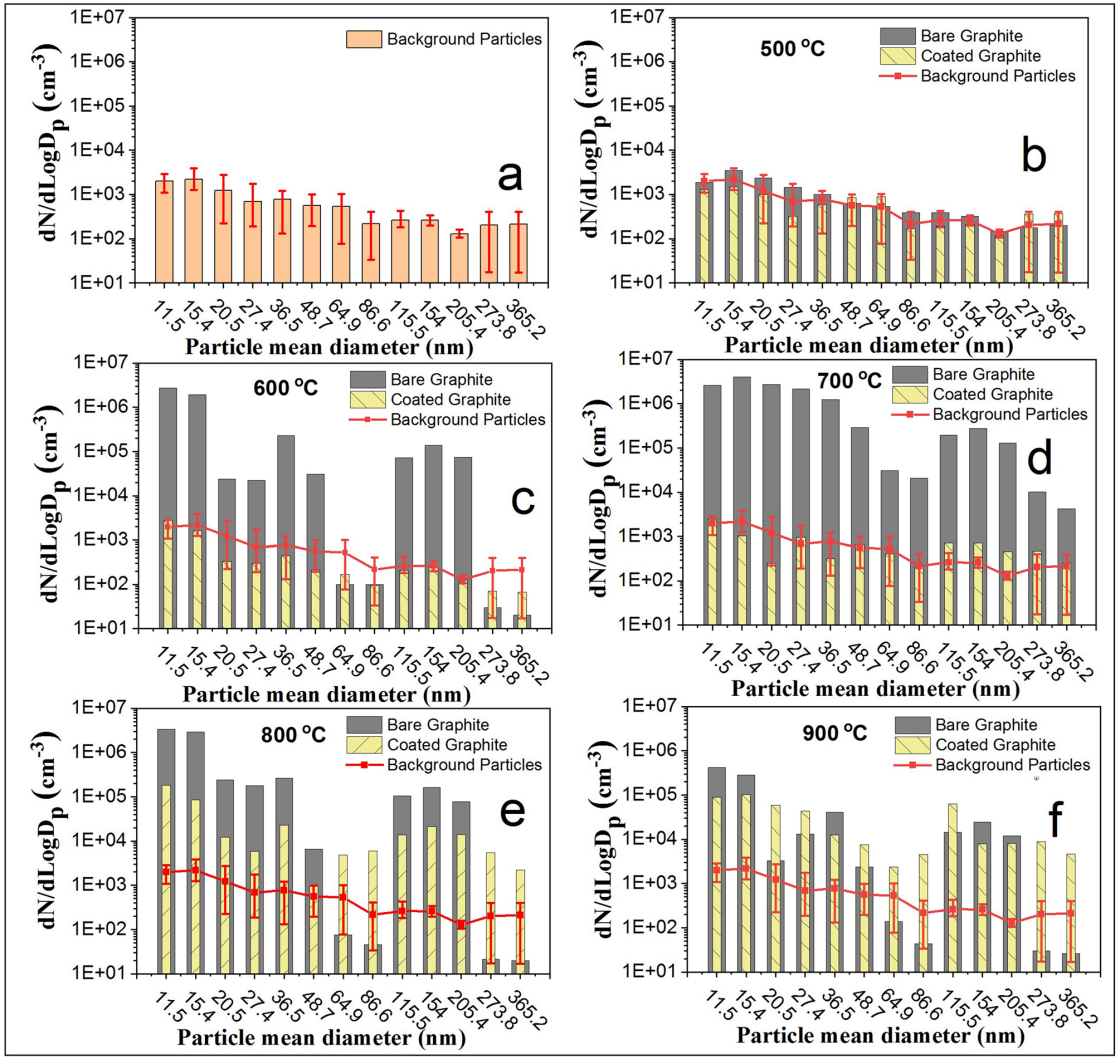
Number size distribution for generated particles from bare and coated graphite specimens.

In the next step, focus was shifted to interpret the number size distribution of the generated particles (wherever applicable) from the oxidized surfaces. Figure 4a, represents the background particle number size distribution (averaged over 2 h) when graphite specimen was not inserted in the tube. This essentially represents the number concentrations corresponding to different mobility diameters as measured by scanning mobility particle sizer. The averaging for other cases was done for the times when the number concentration was higher than the background number concentration value. For the heating temperature of 500 °C (Fig. 4b), number size distribution of the generated particles from bare and coated graphite remained more or less similar as expected. For 600 °C and 700 °C, a difference in terms of particle number size spectrum for bare and coated graphite can be noted (Fig. 4c,d). Whereas number concentration (entire spectrum) was found to be higher than background for the case of bare graphite, readings were in the uncertainty range for the case of coated graphite. As mentioned, coating was successful in cutting off the oxygen supply to graphite surface at temperatures ≤ 700 °C. However, when temperature was raised above 700 °C, increased number concentration was observed for the case of coated graphite (Fig. 4e,f) as well, confirming that graphite oxidation occurred above 700 °C. However as seen in Fig. 3, particle generation continued for the entire oxidation period for bare graphite and was limited to smaller time scales for coated graphite specimens. In addition, some difference in features of number size distribution can also be noted. Whereas particle number concentration was insignificant in 50–100 nm size range (except at 700 °C) for bare graphite, particles in all sizes were observed for coated graphite. For the case of bare graphite, averaged number size distribution representative of graphite oxidation was found to be bimodal consisting of major fraction in the size range of 10–50 nm and 100–200 nm. Number concentration in all size ranges for coated graphite at 800 °C and 900 °C was found to be similar but lesser than that for bare graphite oxidation at 700 °C.

### Gas analysis

**Figure 5:**
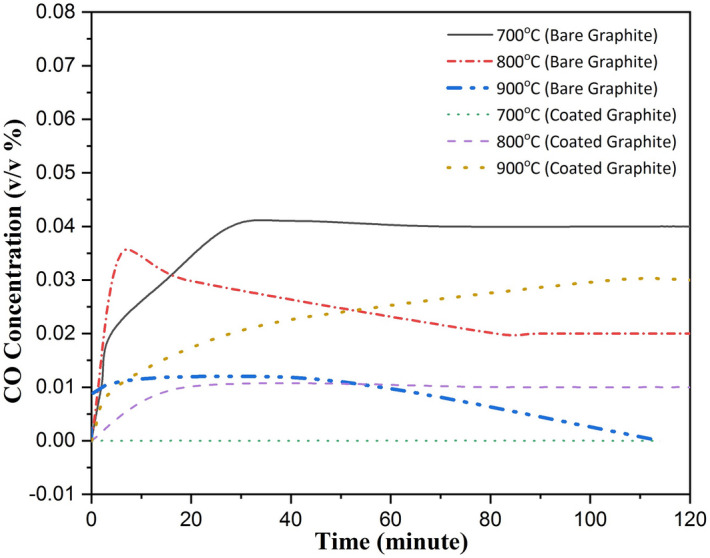
CO generation at different temperatures for bare and Al_2_O_3_ coated graphite.

**Figure 6:**
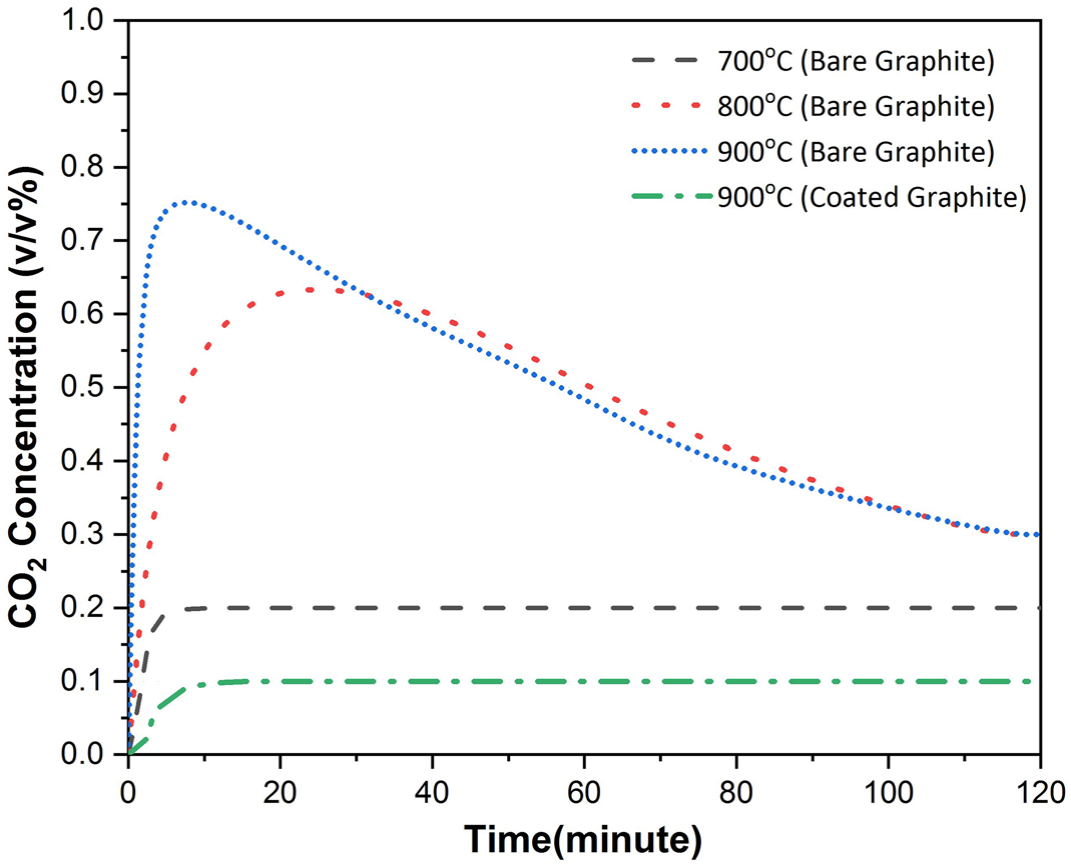
CO_2_ generation at different temperatures for bare and Al_2_O_3_ coated graphite.

Apart from characterizing the oxidation of graphite (bare and coated) with respect to the particle number concentration, temporal evolution of CO and CO_2_ gas concentration was also interpreted. Concentrations of these gases as measured by a gas analyzer are shown in Figs. 5 and 6 as a function of time for different temperatures.

No CO gas was measured by the analyzer for the oxidizing temperatures of 500 °C and 600 °C. For the case of 700 °C, emission of CO gas was observed which was also found to be saturating after ≅ 30 min. CO concentration for this case was highest compared to the oxidation temperatures of 800 °C and 900 °C. Temporal evolution of CO gas concentration indicated that the maximum incomplete oxidation occurred at 700 °C. This is also in agreement with the maximum particle number concentration measured at this temperature (see Fig. 3). However, the transition temperature was found to be higher for the case of coated graphite specimens. For these, CO concentration was not measured by the analyzer till 700 °C. This is due to the reduction of graphite oxidation by inhibiting the influx of oxygen through the coating thickness. For higher temperatures, CO gas concentration was measured by the gas analyzer and was found to be higher for 900 °C as compared to that for 800 °C. At these temperatures, formation of pores leads to the transport of oxygen to the graphite surface resulting in oxidation and the formation of CO. The transition temperature on the basis of CO gas signature was found to be higher for coated graphite specimen. Also, the CO gas concentration at the maximum tested temperature i.e. 900 °C for the coated graphite case was observed to be lesser than the maximum CO gas concentration (at 700 °C) measured for bare graphite. This signifies that the incomplete combustion was lesser for the case of coated specimens even when the oxidation occurred at 200 °C higher temperature than the bare graphite specimens.

These findings were supplemented when the results for CO gas profile were corroborated with those for emission of CO_2_ gas. The temporal evolution of CO_2_ gas concentration for all the cases (bare and coated specimens at different oxidation temperature) has been shown in Fig. 6. For bare specimen, no CO_2_ emission was measured at 500 °C and 600 °C. Afterwards, CO_2_ gas concentration was found to be increasing with the oxidation temperature. However small difference of CO_2_ gas concentration at later times for 800 °C and 900 °C indicates non-dependency of CO_2_ emission on temperature above a threshold temperature. For coated graphite specimens, CO_2_ production started only at 900 °C. It can also be seen that CO_2_ gas concentration for this case was lesser than the minimum recorded concentration for bare graphite. The results based on the temporal profile of CO_2_ emission re-stress the protection of graphite surface against structural degradation due to oxidation at high temperatures, when it was coated with Al_2_O_3_. At 900 °C or higher temperatures, pore formation leads to the increase in oxygen influx to the graphite surface resulting in structural damage even for coated specimens.

### Characterization of residual specimen

#### Weight loss

**Figure 7:**
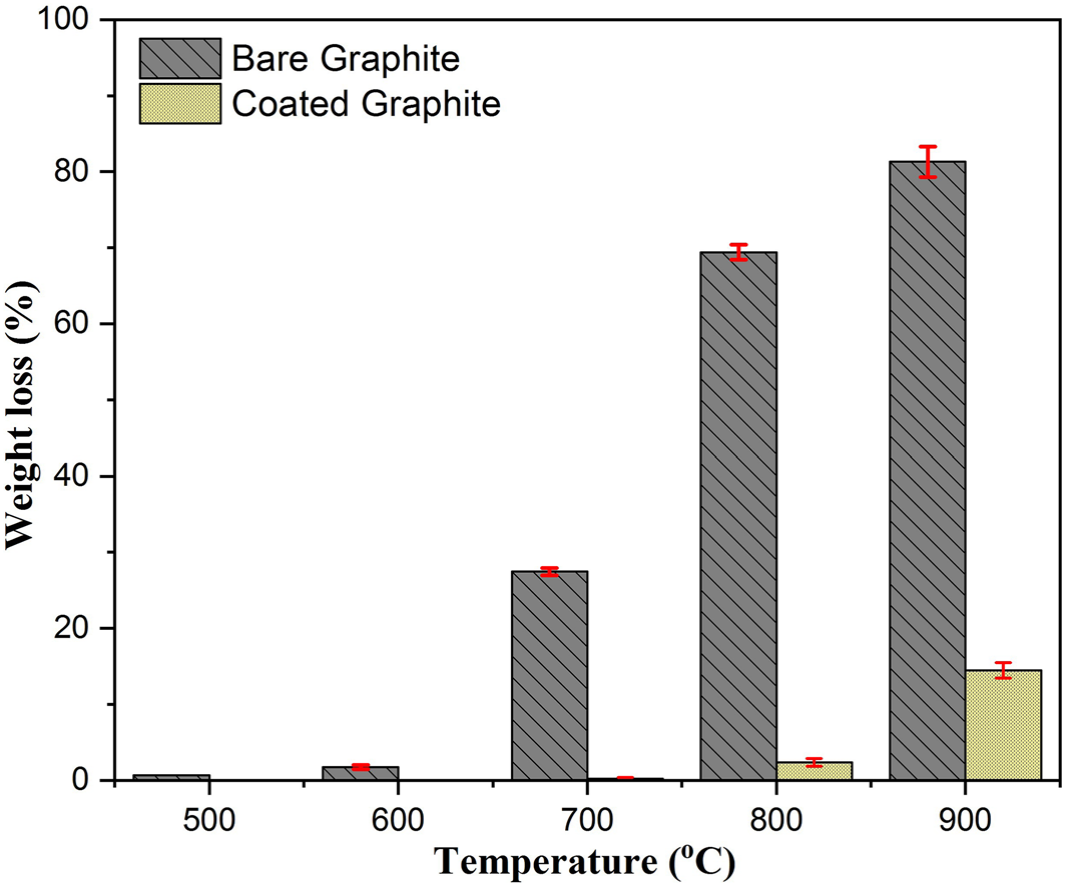
Weight loss percentage in bare and coated graphite specimens after oxidation for 2 h.

After oxidation for 2 h at different temperatures, residual specimens (bare as well as coated) were weighed and the weight loss was calculated for all cases. The weight loss (%) for all cases of experimental test matrix has been plotted in Fig. 7. For bare graphite, weight loss increased with the oxidation temperature leading to almost a reduction of approximately 80% weight of the specimen for 900 °C. However, the rate of increase of weight loss was found to be higher when compared for transition from 700 °C to 800 °C and from 800 °C to 900 °C. For coated graphite specimens, weight loss was negligible even at 800 °C due to the reasons stated above. Weight loss was noted to be approximately 10% at 900 °C for this case. These results are similar to those obtained for nuclear graphite oxidizing in presence of molecular oxygen^30^.

#### SEM analysis of residual specimen

**Figure 8:**
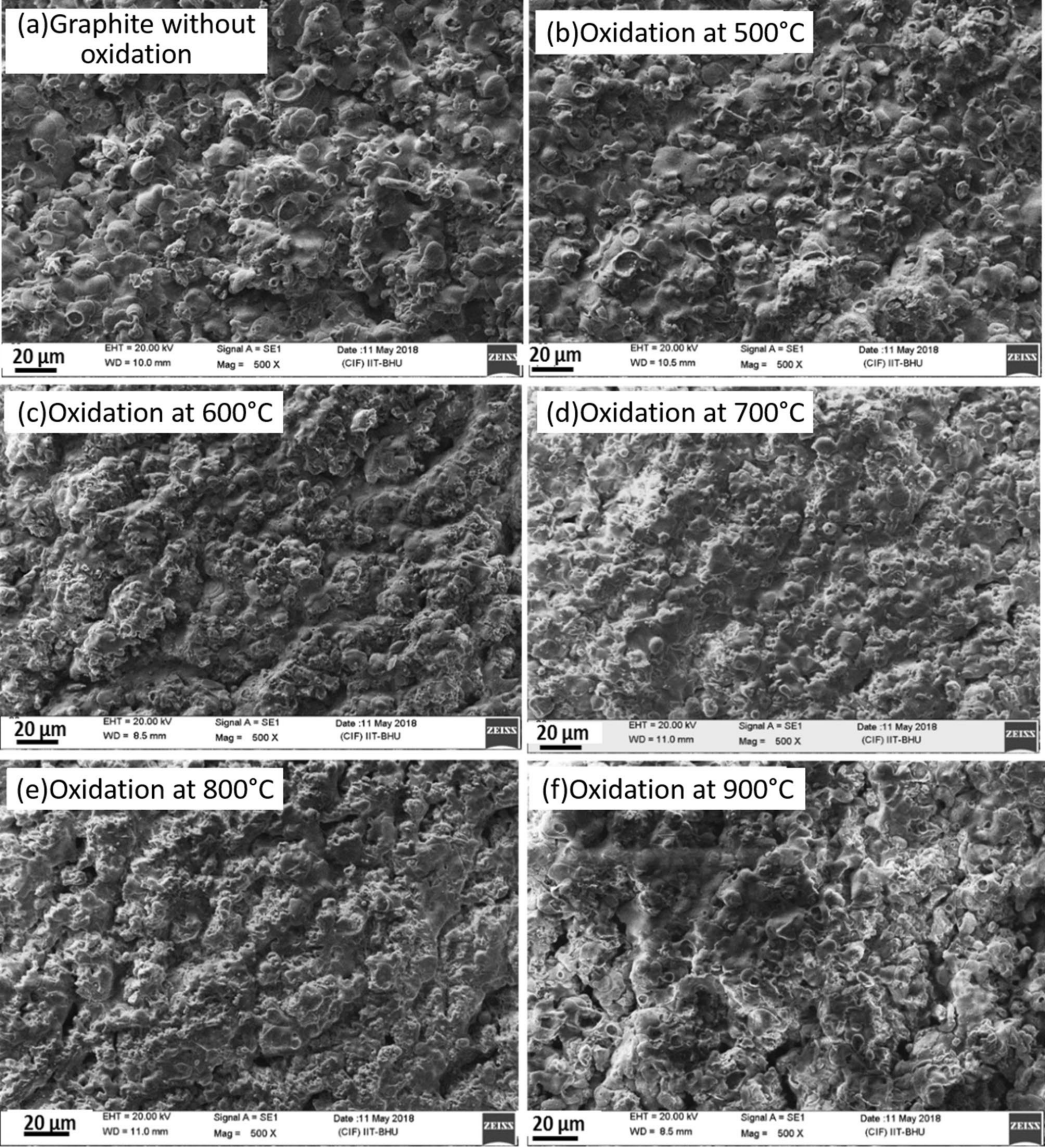
SEM analysis of residual Al_2_O_3_ coated graphite specimens without removing coating layer.

**Figure 9:**
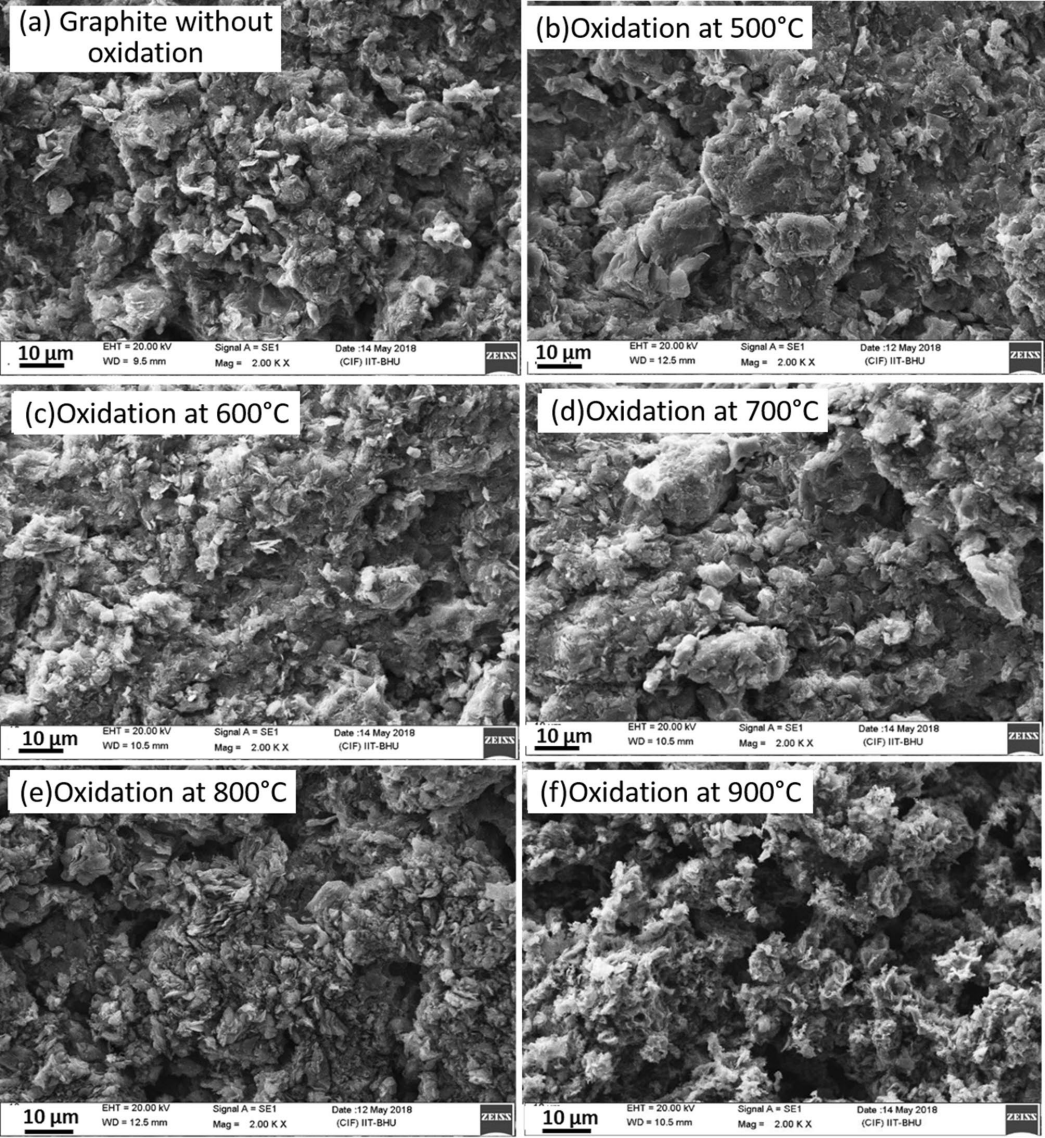
SEM analysis of residual coated graphite specimens after removing coating layer.

In our earlier work^53^, we discussed the effect of oxidation temperature on surface morphology of bare graphite specimens. In that case, the frequency of cavities and flakes increased when the temperature increased from 500 to 600 °C. Beyond 700 °C, formation of flakes reduced which was linked to higher rate of reaction compared to oxygen diffusion^50^. In the present work, Scanning Electron Microscopy (SEM) images were generated using a Scanning Electron Microscope (Make- Carl Zeiss, Model- EVO MA 15/18) to study surface morphological transitions for residual coated graphite specimens. This analysis has been presented for two cases viz. ‘coated residual specimen as such’ and ‘coated residual specimen after removal of coating’. For the latter, hand gloves were used for removal of coating carefully avoiding any surface modifications. Figure 8a–f represents the SEM images of the residual coated graphite specimens without removing the Al_2_O_3_ coating layer. It can be visualized from these plots that no significant change in the microstructure of the coating layer occurred till 700 °C (Fig. 8a–d). Pores were seen to be formed on the coating surface at 800 °C and the frequency increased for 900 °C. Also, the pore size was found to be higher for 900 °C (Fig. 8f) as compared to the case for 800 °C (Fig. 8e). The modifications in the pore characteristics can be linked to the emission of CO and CO_2_ gases and the generation of unburnt particles. Similar analysis performed for the residual specimens after removal of coating can be performed on the basis of the plots shown in Fig. 9a–f. Again, no significant change in surface structure of graphite was seen till 700 °C (Fig. 9a–d). At 800 °C (when pores were seen to be formed on the coating in Fig. 8e), small cavities and flakes were formed on the surface of graphite (Fig. 9e). With further increase in temperature to 900 °C (Fig. 9f), cavity sizes increased and is indicative of an increase in graphite oxidation rate.

## Effect of coating on the generation of particles

The mechanism of oxidation behavior of graphite at high temperature is well documented^32,54,55^. Mechanism for the generation of particles from graphite surface at high temperature was discussed in our previous work performed with bare graphite specimens^53^. Generation of particles signifies the incomplete combustion of the specimen. Above a threshold temperature, graphite starts oxidizing, resulting in morphological modifications of the surface. One of the possible explanation of generation of particles under such conditions is via diffusion limited clustering process^56^. This kind of approach was used in defining source term in place of conventional nucleation term for explosions^57^ and for describing soot nucleation process^58^. In absence of estimation of vapor emission rate, it is difficult to relate thermal quenching to nucleation even when it has been shown to be generating particles when the working temperatures are lower than the phase transition temperatures^59^. Carbon-particle formation in nuclei mode size ranges has also been observed during UV-laser particle-precursor photosynthesis^60^, thermal decomposition of carbon sub oxides at high temperature^61^ and experiments with C_3_O_2_ behind shock waves^62^. However, the experimental conditions of such processes are too restrictive in terms of the present context. The evolution of number size distribution of generated particles could be due to aerosol dynamical processes (mainly coagulation and condensation). In comparison to bare graphite, coating inhibits the oxidation induced degradation and the generation of particles occurs at higher temperatures (see Fig. 3). Even for temperatures of 800 °C and 900 °C, generation of particles takes place sporadically at shorter time scales. It is different from the case with bare graphite where the generation of particles continues for long times as well.

At low temperatures (slow chemical reactions), coating layer acted as a barrier for the penetration of oxygen into the graphite surface. No appreciable weight loss till 700 °C (Fig. 7) indicates that Al_2_O_3_ coating protected graphite from oxidation resulting in negligible particle generation (Fig. 3a–d). The same was also confirmed via SEM images of residual specimens (with and without coating) where frequency of cracks, flakes and pores was seen to be less. CO gas emission, an indicator of incomplete combustion could be measured only at 800 °C. Particle generation for approximately 20 min was also observed at this temperature. However, graphite oxidation possibly gets affected by the formation of a film layer around the specimens, thus stopping the generation of particles as well. Thickness of this layer is expected to be a function of graphite surface temperature. Oxygen at this temperature first diffuses into the coating layer and then through the gas film formed during the oxidation. The frequency as well as the dimensions of the structural damage increased when the temperature was increased to 900 °C (Fig. 8e–f). For this temperature, increased concentration of CO and CO_2_ gases indicates more vigorous combustion (Figs. 5 and 6). Maximum rate of incomplete combustion (CO concentration) and subsequently maximum particle number concentration was observed at 900 °C.

## Limitations of the study

The present study focused on studying the number emission characteristics of particles generated from bare and coated graphite specimens exposed to high temperature conditions. The experimental findings could not be validated with plausible theories due to the limitations of the set-up in terms of the measurements of crucial model parameters. These include the vapour (source) emission rate, chemical composition/speciation of the particles and the shape characterization of generated particles. The gaseous measurements in the present context was limited to CO and CO_2_, and did not focus on other carbon sub-oxides having favorable properties for nucleation. Therefore, the approaches mentioned in the above explanations i.e. diffusion limited clustering growth model and thermal quenching induced nucleation and growth remain to be tested. For the existing or extrapolated test matrix, conditions where alternative particle generation mechanisms such as catalytic cracking could play a role, needs to be probed as well. Although the shifting of transition temperature was noted for the coated graphite, more details on the particle emission characteristics could not be obtained due to the working range of furnace temperature.

## Conclusions

The present work studies the behavior of bare and Al_2_O_3_ coated graphite specimens in terms of particle generation at high temperatures (500–900 °C). For bare graphite specimen, particle formation started at 600 °C. Concentration of emitted CO gas and the particles were observed to be a maximum at 700 °C, decreasing thereafter. Both of these are the products of incomplete combustion indicating that the intensity of oxidation is highest at this temperature. The size distribution of the emitted particles was observed to be bimodal consisting of major fractions in 10–50 nm and 100–200 nm size ranges. For coated specimens, graphite surface was protected against oxidation till 700 °C. At higher temperatures (800 °C and 900 °C), surface degradation initiates, increasing the rate of oxygen diffusion. However, the rate was found to be slower as compared to the case of bare graphite. Transition temperature for the case of coated graphite was found to be higher than that for bare graphite. In contrast to the emissions from bare graphite specimens, particle formation was found to be temporarily sporadic. Interpretation of the effect of Al_2_O_3_ coating on heated graphite in terms of particle generation has been attempted. The findings supplement the oxidation linked database relevant to the performance of graphite at high temperature conditions.
